# P-599. If You’re On-time, You’re Late: Early Detection of Common Respiratory Pathogens Using Wastewater Genomic Surveillance to Shape Hospital Preparedness

**DOI:** 10.1093/ofid/ofaf695.812

**Published:** 2026-01-11

**Authors:** Marleen M Welsh, Benjamin Knisely, Diego Insausti, Tisza A S Bell, Paige Salerno, Valerie J Morley, Dawn Gratalo, Casandra Philipson, Beth Higa Roberts, Gibran J Pierluissi-Jovet, Nora Watson, Michael Backlund, Tyler Moeller, Melissa Austin, Paige E Waterman, Wesley Campbell

**Affiliations:** Booz Allen Hamilton, Bethesda, Maryland; Booz Allen Hamilton, Bethesda, Maryland; BOOZ ALLEN HAMILTON, MIAMI SPRINGS, Florida; Booz Allen Hamilton, Bethesda, Maryland; Ginkgo Bioworks, Andover, MA; Ginkgo Biosecurity, Albuquerque, New Mexico; Ginkgo Bioworks, Andover, MA; Ginkgo Bioworks, Andover, MA; Ginkgo Biosecurity, Albuquerque, New Mexico; National Capital Consortium, Rockville, Maryland; Walter Reed National Military Medical Center, Bethesda, Maryland; WRNMMC, Bethesda, Maryland; Naval Medical Research Unit 6, Maryland, Maryland; Walter Reed National Military Medical Center, Bethesda, Maryland; USUHS, Bethesda, Maryland; Walter Reed National Military Medical Center, Bethesda, Maryland

## Abstract

**Background:**

Wastewater (WW) surveillance (WWS) offers a promising approach to early detection of pathogens. This study explores the relationships between common respiratory viruses detected in WW and clinical laboratory data, examining whether WW pathogen changes are a leading indicator of changes in respiratory disease in hospitals.

**Figure:**
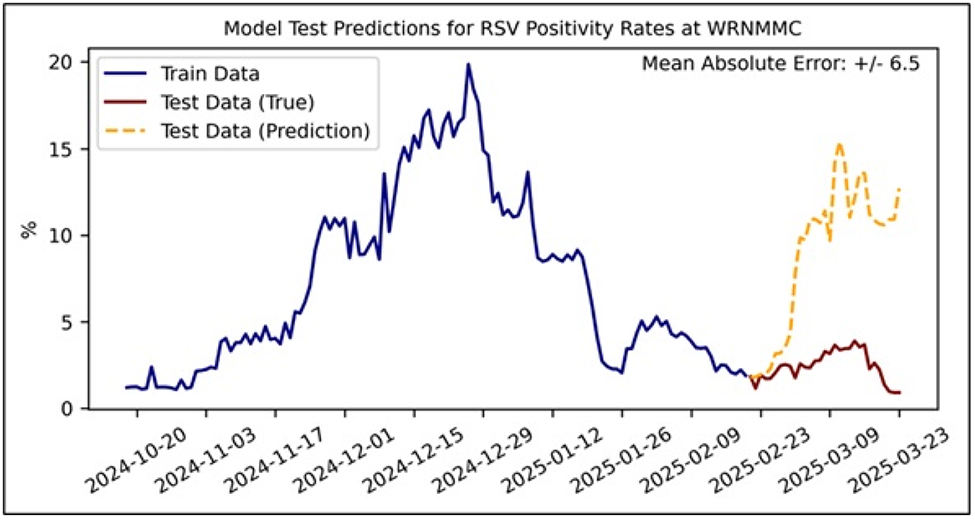

**Figure:**
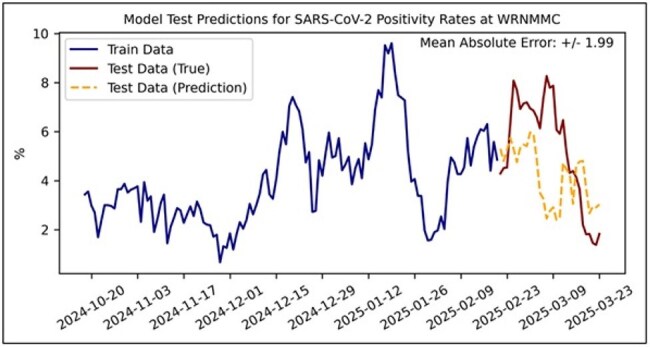

**Methods:**

WW samples from 8 locations at Walter Reed National Military Medical Center were collected 3 times per week from October 2024 to March 2025. SARS-CoV-2, respiratory syncytial virus (RSV), and influenza (flu) A were quantified in the samples using digital polymerase chain reaction (dPCR). Daily percent positivity was calculated from de-identified WRNNMC laboratory results data, and cross-correlations between percent positivity and WW dPCR were assessed. Several modeling approaches were explored, including linear regression (lasso, ridge), tree-based methods (random forests, XGBoost), and neural networks. Further, multiple prediction horizons were examined (1, 3, 5, 7, and 14 days). Train and test sets (80/20%) were constructed from the data. Time-series cross-validation was implemented to evaluate feature selection, models, and model parameters. Mean average error (MAE) between true and predicted positivity rate was used to evaluate model performance.

**Figure:**
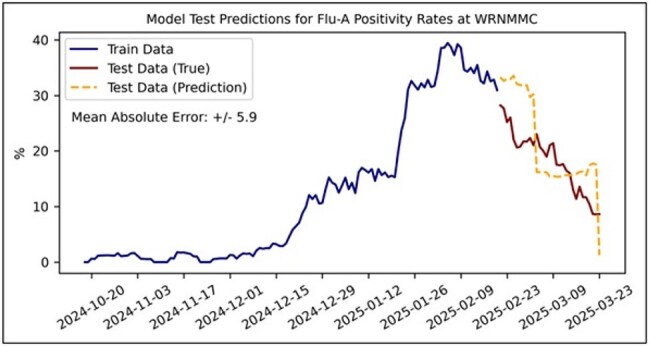

**Results:**

Cross-correlation analyses between laboratory test results and WW dPCR showed very weak correlation for RSV (Figure 1). Weak-moderate correlations were observed for SARS-CoV-2 and Flu A, particularly with shorter time lags for Flu A (Figure 2-3). For forecasting, XGBoost was the best performing model for all pathogens. SARS-CoV-2 models exhibited the best performance across time horizons (MAE: 1.9%-2.8%). RSV and Flu-A models demonstrated less predictive capabilities across varying prediction horizons (MAE: 4.3-11.3% and 4.3%-9.5%, respectively). Further analyses with more time-series data, additional model features, and model optimization are required.

**Conclusion:**

These preliminary findings support that SARS-CoV-2, RSV, and Flu A WW pathogen data may be predictive of clinical outcomes. As additional data are collected, relationships between WW pathogen levels and clinical outcomes will be further explored.

**Disclosures:**

Marleen M. Welsh, Ph.D., Altria Group Inc: Stocks/Bonds (Public Company)|Johnson & Johnson: Stocks/Bonds (Public Company)|Merk & Co Inc: Stocks/Bonds (Public Company)|Pfizer Inc: Stocks/Bonds (Public Company)|Solventum Corp: Stocks/Bonds (Public Company) Valerie J. Morley, PhD, Ginkgo Bioworks: employee|Ginkgo Bioworks: Stocks/Bonds (Public Company) Dawn Gratalo, MS, Ginkgo Bioworks: Stocks/Bonds (Public Company) Casandra Philipson, PhD, PhD, Ginkgo Bioworks: Stocks/Bonds (Public Company)

